# Navigating through apertures: perceptual judgements and actions of children with Developmental Coordination Disorder

**DOI:** 10.1111/desc.12462

**Published:** 2016-10-16

**Authors:** Kate Wilmut, Wenchong Du, Anna L. Barnett

**Affiliations:** ^1^ Perception and Motion Analysis Lab Department of Psychology, Social Work and Public Health Oxford Brookes University UK; ^2^ Division of Psychology Nottingham Trent University UK

## Abstract

Passing through a narrow gap/aperture involves a perceptual judgement regarding the size of the gap and an action to pass through. Children with DCD are known to have difficulties with perceptual judgements in near space but whether this extends to far space is unknown. Furthermore, in a recent study it was found that adults with DCD do not scale movements when walking through an aperture in the same way as their peers. The current study, therefore, considered perceptual judgements and motor behaviour of children with DCD while looking at or walking through apertures. Twenty‐nine children with DCD and 29 typically developing (TD) children took part. In Experiment 1, participants completed a perceptual task, where they made passability judgements. Children with DCD showed a significantly smaller critical ratio (aperture size at which a participant first rotates the shoulders to pass through) compared to their TD peers. In Experiment 2, participants completed an action task where they walked through the same apertures. Children with DCD showed a significantly larger critical ratio than TD peers when body size alone was accounted for. Taken together these results suggest that perception within a static context is different from that within a dynamic context for children with DCD. However, despite this difference we have demonstrated a clear relationship between perception and action in children with DCD. A video abstract of this article can be viewed at: https://youtu.be/SABXFrAJtF8

## Research highlights

- Children with DCD underestimated the space they need to pass when making a perceptual judgement.
- Perceptual judgements regarding absolute size were not different across groups with and without DCD.
- Children with DCD overestimated the space they need to pass when executing a movement.
- A relationship exists between perception and action in children with DCD.

## Introduction

As we move around the environment we encounter many obstacles such as parked cars, pedestrians and street furniture, and navigating passed these often involves judging whether a gap is large enough to fit through. This requires the ability to visually estimate the size of the gap, integrate accurate information regarding body size and then determine whether the gap is large enough to fit through either with or without a shoulder rotation. Once this judgement has been made, we need to execute a movement which allows safe passage. Misjudgement in our perception of passability or in the execution of an adaptive movement to pass through may result in collision/injury. The constraints‐based approach to understanding motor behaviour (Newell, 1986) integrates ideas from dynamical systems theory (Thelen, 1989) and ecological psychology's direct perception (Gibson, 1979) and would suggest that a motor response *emerges* as a function of the perception of the environment and what it affords. Affordances are tied to the interaction between the physical properties and capabilities of the actor and the physical properties of the environment (Gibson, 1979). A decision to rotate the shoulders when passing through a gap depends on the perception of the affordances of the gap, i.e. the perception of the gap in relation to body size and action capabilities. The constraints‐based approach also states that any motor response is constrained and influenced by the task, the environment and the individual (Keogh & Sugden, 1985; Newell, 1986). Therefore, the response to walking through a gap emerges from demands of the task (e.g. the size of the gap), the environment (e.g. stability of the gap) and the individual (e.g. their ability to determine affordances and their ability to control movement). Central to this approach is the link between perception and action (Sugden & Wade, 2013).

When considering passage through an aperture Warren and Whang (1987) found that participants rotated their shoulders for apertures less than 1.3 times shoulder width regardless of body size (this is termed the ‘critical ratio’). Warren and Whang (1987) concluded that movement behaviour is influenced by knowledge of anthropomorphic measures, i.e. the perception of affordances is based on one's own body size. Research studies considering typical children (Wilmut & Barnett, 2011), the elderly (Hackney & Cinelli, 2011) and adults with motor difficulties (Wilmut, Du & Barnett, 2015) have also shown that participants rotate the shoulders based on body size. However, Wilmut and Barnett (2011) and later Wilmut *et al*. (2015) have shown that the degree to which a mover rotates his/her shoulders is also based on movement variability; as movement variability increases the degree of shoulder rotation also increases. This seems to be an adaptive strategy which allows participants to tailor the safety margin (distance between the shoulders and the edge of the door) to their own movement ability (Wilmut *et al*., 2015). The studies described thus far have measured participant *behaviour* at an aperture, which could be influenced by both one's ability to perform a movement and one's ability to judge the affordances of the environment. Other studies have considered the judgement of passability outside a movement context. Warren and Whang (1987) found that participants can make consistent perceptual judgements regarding passability when viewing from a distance and that disrupting the ratio between standing height and eye height without an observer's knowledge impairs their ability to make passability judgements. The scaling of visual scene to eye height is clearly established in Gibson's theory of direct perception (Sedwick, 1973; Gibson, 1979). In a later study Higuchi, Takada, Matsuura and Imanaka (2004) concluded that when making passability judgements, participants tend to over‐estimate the space needed for them to pass without turning. However, this ‘over‐estimation’ was not greater than that seen when participants actually passed through an aperture in Warren and Whang's study; therefore, these participants may simply be perceiving passage on the basis of the space they would actually need to walk through.

Developmental Coordination Disorder (DCD) describes a condition in which motor coordination is below the level expected for an individual's age. Almost 2% of children in the UK present with DCD (Lingam, Hunt, Golding, Jongmans & Emond, 2009), displaying fine and gross motor difficulties (Sugden, 2006) which persist into early adulthood, continuing to have a negative impact on everyday life (Kirby, Edwards, Sugden & Rosenblum, 2010). Anecdotal evidence from parents of children with DCD and the professionals working with them suggests that they are prone to colliding with obstacles in their pathway (Geuze, 2007). These navigation difficulties may be due to a range of factors: a visual perceptual deficit; a difficulty with integrating knowledge about body size into a passability judgement; difficulty executing the necessary movement; or a lack of awareness of movement variability.

Historically, DCD has been considered within an information processing framework, i.e. one of indirect perception (Sugden & Wade, 2013) and specific visual perceptual deficits have been reported. However, the tasks considered within this framework are limited as many studies considered visual perceptual ability in the absence of action. For example, research has highlighted poor visual discrimination ability (Henderson, Barnett & Henderson, 1994; Hulme, Biggerstaff, Moran & McKinlay, 1982a; Hulme, Smart & Moran, 1982b; Hulme, Smart, Moran & McKinlay, 1984) and poor performance on visual perceptual tests used in clinical settings (Tsai, Wilson & Wu, 2008) in children with DCD. However, there seems to be no straightforward relationship between perception and motor deficits in these children (Henderson *et al*., 1994) and so there has been considerable debate about the extent to which poor visual perceptual skills may explain the motor difficulties of children with DCD. Drawing on a more contemporary framework, the constraints‐based approach provides a more useful way of investigating motor deficits in these children. This approach advocates the need to consider perception within the perception–action context and that the task, the environment and the individual are all possible constraints on a motor response. Furthermore, this broad approach can also encompass information‐processing accounts of motor performance (see Anson, Elliott & Davids, 2005). Within this context a series of studies have considered the ability of children with DCD to judge action capabilities. Here children are making perceptual judgements regarding action ability and therefore the task is embedded into the perception–action cycle. These include the judgement of vertical reaching height and sitting height (Johnson & Wade, 2007), horizontal reaching (Johnson & Wade, 2009) and maximum sitting height with standing height artifically altered (Chen, Tsai & Wu, 2014). In all of these studies children with DCD made less accurate judgements of action capability compared to their peers, with no clear pattern of over‐ or under‐estimation. From these findings it was suggested that DCD may be associated with a deficit in the sensitivity to the fit between their own body and the environment (Wade, Tsai, Stroffregen, Chang & Chen, 2007).

In terms of how children with DCD adapt their movements to avoid obstacles there is a paucity of data. Deconinck, Savelsberg, De Clercq and Lenoir (2010) did consider the nature of approaching and stepping over an obstacle in a group of children with DCD. Although the children with DCD were able to adapt their gait, they exhibited difficulty controlling momentum due to the increased balance demands. Furthermore, in a previous study adults both with and without DCD were asked to walk up to and through a series of apertures scaled to body size (Wilmut *et al*., 2015). Adults with DCD scaled their decision to rotate their shoulders on the basis of both body size and movement variability, while TD participants based the decision on body size alone. In terms of adaptation at the aperture the adults with DCD slowed earlier in the approach and to a greater extent when a shoulder rotation was required. This demonstrates a pronounced difference in the way in which movement is adapted in this population. To date no studies have considered action judgements in an aperture navigation task in children with DCD.

The current studies aim to consider the perception of affordance and actual passage through an aperture in children with DCD. An information‐processing account of DCD would specify that the motor problems arise from a visual perceptual deficit. However, although perceptual deficits are reported, the relationship with poor motor performance has not been established (Henderson *et al*., 1994). The previous work of Johnson and Wade (2007, 2009) would suggest a difficulty in the perception of affordances in these children; however, motor performance on the same tasks was not measured and so it is difficult to determine the relationship between perception and action from these studies. Interestingly some studies have considered the relationship between perceptual judgements and movement control. For example, Chen *et al*. (2014) found that the TD group, but not the DCD group, showed a relationship between sway and perceived sitting height, whereby less sway was correlated with a more accurate judgement. The authors conclude a difference in the perception–action coupling of children with and without DCD. Although this study goes some way to consider the relationship between perception and action in children with DCD, the difference between the perceptual task and the movement actually measured makes it difficult to fully understand this relationship. Chen and Wu (2013) did also include correlations between perception and action; however, as they considered the TD and DCD group together in one correlation this tells us very little about the relationship in children with DCD compared to their peers.

## Experiment 1: Visual judgements

In Experiment 1 we considered the ability of children with DCD to make both absolute visual estimations of size and their ability to make passability judgements. Research studies suggest that children with DCD have difficulty making absolute size judgements using a range of table top tasks (Henderson *et al*., 1994; Hulme *et al*., 1982a; Hulme *et al*., 1982b; Hulme *et al*., 1984; Tsai *et al*., 2008). However, these studies have only considered judgements in near space (i.e. within reaching distance). This is certainly relevant for the performance of manual skills where objects are reached for and manipulated. However, locomotor skill involves processing of visual information from far space (i.e. out of reaching distance) and previous work has demonstrated that visual information in far space may be processed differently from that in near space. For example, Weiss *et al*. (2000) looked at neural processing during a line bisection task and a pointing task in near versus far space using PET (Positron Emission Tomography). They found greater neural activity in the dorsal visual motor stream (dorsal occipital cortex and parietal cortex) when processing in near as compared to far and a greater activity in the ventral visual perceptual stream (ventral occipital cortex and right medial temporal cortex) when processing in far versus near space (Weiss, Marshall, Wunderlich, Tellmann, Hallisan *et al*., 2000). One study which plausibly considered visual judgements of size in children with DCD in far space is that by Chen and Wu (2013) who demonstrated a difficulty with the perception of absolute size in far space in children with DCD. However, Chen and Wu's (2013) task required participants to process information in both near and far space and thus any deficit is not clearly isolated to one or the other. Therefore, whether these children would have difficulties with perceptual judgements in far space is unclear. In the current study we considered whether children with DCD could determine the point at which two apertures, 7 m away, were of the same size. Given that previous research studies have suggested that children with DCD struggle with absolute size estimates in near space we expected to see less accurate judgements of absolute size in the children with DCD compared to the typically developing children. In line with previous studies we expected these inaccuracies to show up in terms of higher absolute error, but no clear pattern of over‐ or under‐estimation in size. Absolute size judgements provide some information about visual perceptual skills in far space, but do not tell us anything about whether an individual can use information regarding their body size and make an accurate passability judgement. In the second part of Experiment 1 we therefore considered the point at which children with DCD stated that they would need to rotate their shoulders to pass through an aperture presented in far space. Previous research focusing on action judgements in children with DCD has suggested that they are not able to make these as accurately as their peers (Johnson & Wade, 2007) and so in this study we would expect to find less accurate judgements in the participants with DCD compared to the typically developing participants. This will be apparent in terms of a higher degree of absolute error but again no clear under‐ or over‐estimation. Critical ratio will be calculated, but given the lack of over‐ or under‐estimating pattern of error no difference is expected between the groups in terms of critical ratio.

### Method

#### Participants

This project was approved by the Oxford Brookes University Research Ethics Committee. Twenty‐nine participants with DCD (aged from 7 to 17 years) and 29 age (to within 6 months) and gender matched typically developing individuals were recruited for this study. Details regarding these participants can be found in Table 1. Participants with DCD were recruited from two sources: a group known to the authors from previous studies and a local support group for individuals with DCD and their families. All participants with DCD were assessed and selected in line with the DSM‐5 criteria for DCD and with recent UK guidelines (Barnett, Hill, Kirby & Sugden, 2015). For criterion A, the Test component of the Movement Assessment Battery for Children second edition (MABC‐2; Henderson, Sugden & Barnett, 2007) was used to determine motor skill below the level expected for the individual's chronological age. The participants with DCD scored below the 16th percentile on this test. The MABC‐2 Checklist, the DCD‐Q (Wilson, Kaplan, Crawford, Campbell & Dewey, 2000) and a telephone interview with the parent were used to determine that the motor impairment significantly impacted on daily living (criterion B) and that the onset of that difficultly was in early childhood (criterion C). The telephone interview was also used to determine that the difficulties were not due to a known neurological impairment or intellectual disability (criterion D). Parents of the TD participants completed a telephone interview and the MABC‐2 Checklist and DCD‐Q to confirm that no movement difficulties were present.

**Table 1:** Descriptive information for the two cohorts

	TD	DCD	Sig
*N*	29	29	
Mean age (yrs:mo)	11:09	12:01	*ns*
Standard deviation of age	3:16	3:14	*ns*
Age range (yrs:mo)	7:11–17:11	7:8–17:10	*ns*
Gender ratio (F:M)	22:7	22:7	*ns*
MABC‐2 test mean percentile	–	3.13	–
MABC‐2 test percentile range	–	0.1–9	–
MABC‐2 Checklist number of children scoring in lowest category	3	28	*p *<* *.001
DCD‐Q total score	67.9	33.5	*p *<* *.001
Shoulder width (cm)	34.0	33.6	*ns*
Body width (cm)	38.5	39.1	*ns*
BMI	17.6	19.9	*ns*

Given the co‐occurrence of motor and attention difficulties, all parents completed the Strengths and Difficulties Questionnaire (SDQ; Goodman, 1997). We focused on the inattention/hyperactivity subscale and used the classifications specified by the test. Ten of the children with DCD had high or very high scores on this subscale compared to none of the typically developing children. Running analyses both with and without these children did not alter the outcome of the findings and so these individuals were included in the study.

#### Apparatus and procedure

Participants completed two tasks, a visual estimation task where they were asked to judge absolute size and a perceptual action judgement task where they were asked to make passability judgements. All participants completed the visual estimation task followed by the perceptual action judgement task.

##### Visual estimation task

Participants stood 7 m away from two apertures which were created between three partitions (the partitions were 2 m × 1 m in size and consisted of a single piece of wood attached to a triangular base supported by castors). Directly behind the partitions was a curtain which ensured that both apertures had a similar backdrop. A standing distance of 7 m was chosen so as to align this with previous work on adults with DCD where they were asked to pass through an aperture. On a given trial either the aperture on the left or the right was set at a width of 60 cm; this was the standard aperture and did not change in size for the rest of that trial. The other aperture, the non‐standard, started at either 100 cm (decreasing condition) or 20 cm (increasing condition). The participant was asked to state whether the two apertures were the same size or not; if they stated that the apertures were of a different size the non‐standard aperture was decreased (decreasing condition) or increased (increasing condition) by 2 cm. This continued until the participant stated the apertures were the same size. The size of the non‐standard aperture was recorded at this point. Participants completed eight trials, four increasing and four decreasing. The increasing/decreasing conditions were pseudo‐randomized as was the side of the standard. The participant turned around between trials to face away from the apertures.

##### Perceptual action judgement task

**Figure 1:**
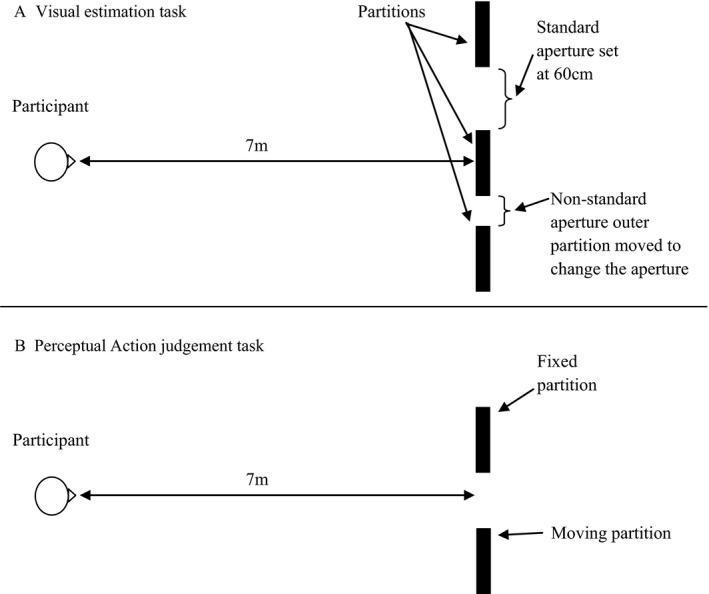
(A) An illustration of the set‐up for the visual estimation task. In the illustration the aperture to the participant's right is the non‐standard and an increasing trial is depicted. (B) An illustration of the perceptual action judgement task, in the illustration the partition to the participant's right is the moving partition.

Participants stood 7 m away from one aperture which was created between two partitions (partitions described previously). Initially shoulder width (distance between the left and right acromion process) and body width (widest point on the upper body) was measured to the nearest mm using digital callipers. On a given trial participants were presented with an aperture that was either 0.9 times their shoulder width (increasing condition) or 2.1 times their shoulder width (decreasing condition). For an illustration of the set‐up see Figure 1. Participants were asked to judge whether they could walk through the aperture presented with or without turning their shoulders. If they judged that they could pass without turning they were to state ‘straight’, if they needed to turn they were to state ‘turning’. Once they had made this initial judgement, the aperture was increased (increasing condition) or decreased (decreasing condition) in size by 2 cm and the participants had to make a new judgement. This continued until the participant switched from a ‘turning’ judgement to a ‘straight’ judgement (increasing condition) or from a ‘straight’ judgement to a ‘turning’ judgement (decreasing condition). The relative size of the aperture was noted at this point. The experiment consisted of six trials, three increasing and three decreasing which were presented in a pseudo‐randomized order. The participant turned around between trials to face away from the apertures.

#### Data analysis

##### Visual estimation task

Error was calculated by comparing the non‐standard finishing size against 60 cm (the standard size): absolute error was calculated as the un‐signed difference between the non‐standard and 60 cm; constant error as the signed difference between the non‐standard and 60 cm and; variable error as the standard deviation of absolute error.

##### Perceptual action judgement

It was important to compare equivalent points for the increasing and decreasing trials. Given that usually the critical ratio is defined as the aperture size at which a participant switches from ‘straight’ (not turning) to ‘turning’, we chose to use this point for all calculations. Therefore, for both decreasing and increasing trials we selected the largest aperture size which the participant stated would require a turn. Both error of the judgement and critical ratio of the judgement were calculated. For judgement error; absolute error was calculated as the un‐signed difference between the aperture size at which the participant indicated that they would first make a turn and body width; constant error as absolute error but using un‐signed differences and; variable error as the standard deviation of absolute error. Critical ratio was calculated with respect to both shoulder width (SA ratio) and body width (BA ratio). Shoulder width is the measure that is typically used when considering passage through an aperture. However, the body can be wider than the shoulders and previous research has shown a greater incidence of raised BMI in a DCD population (Cairney, Hay, Faught & Hawes, 2005). In the current study, we saw no significant difference between groups for shoulder width, body width or BMI; however, a lack of group difference does not necessarily mean that the significant difference we saw between shoulder width and body width [*t*(57) = 15.73 *p *<* *.001] was not more pronounced in some children than in others.

#### Statistical analysis

Given the wide age range of the children included in this study but no clear way to separate these into an older and a younger group, we decided to use ANCOVA with age as the covariate to analyse the data; in each case we state whether we find that age did act as a significant covariate. Significant interactions were followed up using simple main effect tests with a Sidak correction for multiple tests. Significant main effects were followed up using post‐hoc tests once again with a Sidak correction for multiple tests. Where the assumption of sphericity is violated, the Greenhouse‐Geisser correction is reported. Partial eta squared is reported as a measure of effect size and the level of significance was set at 0.05. Relationships between dependent variables were investigated using Pearson's bivariate correlations.

### Results

#### Visual estimation task

The absolute error, constant error and variable error were compared across group using a one‐way ANCOVA (group). Data can be found in Table 2. No significant group or age effects were found for any of these measures (*p *>* *.05).

**Table 2:** Error data for the visual estimation task. Absolute error, constant error and variable error are given in cm for the children with and without DCD. Standard error is given in parentheses

	TD	DCD
Absolute error (cm)	5.37 (3.19)	5.49 (2.38)
Constant error (cm)	1.04 (2.62)	−0.07 (3.07)
Variable error (cm)	6.51 (3.49)	6.73 (2.67)

#### Perceptual action judgement

Absolute, constant and variable errors are displayed in Table 3. One‐way ANCOVA (group) found a significant effect of group for both absolute [*F*(1, 55) = 23.11, *p *<* *.001, η^2^ = .30] and constant [*F*(1, 55) = 24.25, *p *<* *.001, η^2^ = .31] error. In both cases the participants with DCD showed a smaller degree of error compared to the TD participants. No significant group effect was seen for variable error (*p *>* *.05). In terms of critical ratio, both the shoulder and body width critical ratio showed a significant main effect of group [SA: *F*(1, 55) = 8.81, *p *=* *.004, η^2^ = .14, BA: *F*(1, 55) = 18.21, *p *<* *.001, η^2^ = .25]. In each case this was due to a higher critical ratio in the TD participants compared to the participants with DCD. In all cases age was not found to be significant (*p *>* *.05). Data can be found in Table 3.

**Table 3:** Absolute error, constant error, variable error and critical ratios for the perceptual action judgement task

	TD	DCD
Absolute Error (cm)**	14.51 (4.2)	8.70 (4.94)
Constant error (cm)**	14.49 (4.19)	7.88 (5.81)
Variable error (cm)	4.21 (1.68)	4.03 (1.66)
Critical ratios		
SA*	1.54 (0.14)	1.41 (0.19)
BA**	1.39 (0.10)	1.22 (0.18)

**p <* *.001; *p <* *.05.

### Discussion

In a visual estimation task children with DCD showed no difference in absolute error, constant error or variable error compared to their peers. Therefore, there is no evidence from this study that children with DCD have difficulty in making perceptual judgements in far space. This contradicts previous studies in near space which have suggested that children with DCD make more errors than TD children when judging absolute line length (Hulme *et al*., 1982a; Hulme *et al*., 1982b; Hulme *et al*., 1984). One explanation may be that these previous findings relate to tasks performed in near space and may go some way to explain the difficulties children with DCD have with fine motor skills. Given that judgements in far space require different neural systems from those in near space (Weiss *et al*., 2000), it may be that the neural systems involved with perception in far space are intact in individuals with DCD, whereas those involved with perception in near space are impaired. Support for this comes from previous studies which have identified a possible dorsal stream deficit in children with DCD (for example, see Bair, Kiemel, Jeka & Clark, 2012). However, this research is inconclusive and further studies are needed to examine this finding.

In terms of judgements of passability we see a smaller absolute and constant error in the children with DCD compared to their peers. This finding seems to contrast with studies which found less accurate judgements in children with DCD but no clear under‐ or over‐estimation (Johnson & Wade, 2007, 2009; Chen *et al*., 2014). A smaller error, as demonstrated in the participants with DCD, suggests that they judge a need for less relative space, i.e. less of a safety margin. This is reflected in the critical ratios, with the participants with DCD showing a smaller shoulder and body width critical ratio. It is difficult at this point to state exactly what this means. It would seem that the children with DCD are more ‘accurate’ at making these judgements as indicated by their smaller error. However, as stated previously, passing through an aperture is part of an ongoing movement and as such a safety margin is needed in order to avoid collision. Therefore, a lower critical ratio doesn't necessarily mean a ‘better’ judgement. It may be that the children with DCD are under‐estimating how much space is needed for them to safely pass through or that they naturally leave a smaller safety margin when passing through apertures compared to their peers and that their judgements as measured in this experiment are in line with their behaviour. In the next experiment we ask the same participants to walk up to and pass through a series of apertures while we measure their movements.

Intriguingly this first study suggests that children with DCD make judgements of absolute size in far space which are in line with their peers but they make affordance judgements which are very different from their peers. This finding suggests that, at least in far space, the mechanisms behind the group differences in judgements of action capabilities are not due to a generalized deficit in visual size perception but are rather more subtly linked to judgements regarding body fit / size. The perception–action model proposes that there is one visual stream which encodes visual information for perception and another which encodes visual information for action (Milner & Goodale, 1995). Although there is some debate in the literature as to whether this is too simplistic, it is generally accepted that this does capture some aspects of visual processing (see Schenk & McIntosh, 2010, for a review). This model proposes the use of the ventral stream for vision related to perception only and the dorsal stream for vision related to action. The two tasks that we presented in Experiment 1 can be thought of in terms of visual processing; the first task, judgement of absolute size, is a perception only task while the other involves some aspect of an intention to act. Thus it may be that the children with DCD can process perceptual information in far space for perceptual judgements, but then have difficulty with processing visual information for action, i.e. a difficulty with dorsal stream functioning. Previous studies have reported deficits in both the dorsal and ventral stream (Sigmmundsson, Hansenc & Talcott, 2003; Tsai *et al*., 2008) which seems to contradict this finding. However, given that we have stated previously that visual information in far space may only be processed by the ventral stream and not the dorsal stream it may also be that when in far space the ventral stream processes ‘action’ information and does this inefficiently in children with DCD. Studies designed to consider this are needed before we can confirm these conclusions.

## Experiment 2

In Experiment 2 we considered the movement behaviour at the doorway in the same group of children with DCD as described in Experiment 1. The constraints‐based approach states that action emerges from self‐organized movement patterns which are constrained (and influenced) by the task, the environment and the child. In Experiment 1 we have demonstrated that children with DCD perform differently from their peers on a ‘non‐motor’ perceptual task relating to action capabilities. In Experiment 2, we plan to build on this and add a motor component to the task. The methodology used here is the same as described in a previous publication (Wilmut *et al*., 2015) and allows us to measure the behaviour of a participant while approaching and passing through a series of apertures scaled to shoulder width. Wilmut *et al*. (2015) concluded that the movement adaptations and the scaling of critical ratio in adults with DCD was an adaptive strategy which allowed them more time to make an adjustment and which allowed for a greater safety margin, both of which helped to avoid a collision. However, our findings from Experiment 1 suggest that children with DCD judge that they can pass through narrower apertures than their peers. This may explain the anecdotal evidence that these children are prone to bumping into objects. The current study examines whether the passability judgements seen in Experiment 1 are in line with behaviour at an aperture or whether children with DCD adopt a different strategy. In terms of the critical ratio, Experiment 1 would suggest that the children with DCD will show a significantly lower critical ratio than their peers; however, the previous study on adults with DCD suggests that they would show a significantly larger critical ratio than their peers; which of these we find remains to be seen. In terms of the movement adaptations at the door we expect to see similar adjustments in the children with DCD as seen previously in adults with DCD; so therefore, a reduction in speed which occurs earlier in the movement and which is greater than that seen in the typically developing children. We also expect to see a higher degree of shoulder rotation at the door which is related to their movement variability.

### Method

#### Participants

These were the same as described in Experiment 1

#### Apparatus

Two partitions, as described in Experiment 1, were used to create an aperture of 7 m in front of the participant. A 16 camera Vicon motion capture system running at 100 Hz was used to track the movement of three 9.5 mm spherical reflective markers placed on the left and right acromion process (LAP and RAP) and on the seventh cervical vertebrae (C7). In order to determine the point at which a participant passed through the aperture, two additional markers were placed on the inner edge of each partition.

#### Procedures

The shoulder width measured in Experiment 1 was used to calculate the seven shoulder to aperture (SA) ratios (0.9, 1.1, 1.3, 1.5, 1.7, 1.9 and 2.1). On each trial the participant was asked to stand 7 m away from the aperture and to focus on a spot on the floor in front of them. On the initiation of a trial the participant was instructed to look up and walk at a self‐selected speed towards and through the aperture to the stop point located 2 m passed the aperture. Movement was captured once the participant was within 4 m of the aperture up until the point of passing the aperture threshold. As the participant walked around the side of the partitions and back to the start point, an experimenter changed the size of the aperture ready for the next trial. The participant was instructed not to look up at the aperture until instructed to do so. Once each trial had started, a second experimenter moved the start point ±20 cm in the anterior‐posterior direction in order to prevent a consistent start point. Prior to the start of data collection the experimenter demonstrated walking through a wide and a narrow aperture. Although no specific instructions were given on when to turn, the demonstration clearly showed the experimenter turning to fit through the narrow aperture. To ensure understanding, the younger children (<12 years) were also given the opportunity to practice walking through both a wide and a narrow aperture, this was not deemed necessary for the older children (>12 years). The order of SA ratios was pseudo‐randomized so that no one SA ratio appeared more than once on consecutive trials and so that there was no predictable increase or decrease in SA ratios. Each aperture ratio was presented five times (35 trials per participant) in one of two of these pseudo‐randomized orders.

#### Data analysis

All participants successfully passed through each aperture without colliding with either partition. Vicon movement data were filtered using an optimized low pass Woltring filter with a 12 Hz cut‐off point and then analysed using tailored matlab routines. Actual aperture width was determined using the medio‐lateral positions of the doorway markers and then compared to the desired aperture size; this was found not to deviate more than ±8.19 mm; this value was deemed small enough to be negligible. Kinematic variables were taken across two phases of movement: (1) the approach phase, which was defined as the first 2 seconds of movement (2 seconds was used as we saw no adjustments to movement prior to this point); and (2) the crossing phase, which covered anything from the first 2 seconds up until the point of passing the aperture threshold.

##### Shoulder angle (^o^)

This was calculated as the yaw of the shoulders. *Baseline yaw:* mean angle of yaw across the approach phase. *Shoulder angle at the aperture:* yaw as C7 passed the partitions. *Variability of shoulder angle at the aperture:* Standard deviation of yaw angle at the aperture.

##### Speed

A trend line was fitted to movement speed and all subsequent measurements were taken from this line. *Approach speed (ms* ^*−1*^ *)*: average movement speed across the approach phase. *Reduction in speed (ms* ^*−1*^ *):* a reduction in speed was defined as when speed after approach dropped more than 3*SD* below the approach speed; if this happened on a given trial then ‘reduction in speed’ was the difference between approach speed and resulting speed. If no apparent reduction in speed was seen then this was set to a value of 0 ms^−1^. *Time after initiation of the reduction in speed (ms):* If there was a reduction in speed the time between this reduction and the point at which the participant passed the aperture threshold was calculated.

##### Trunk movement (mm)

*Lateral trunk movement:* average lateral movement of C7 across the approach phase. *Lateral trunk movement variability:* Standard deviation of the lateral trunk movement within each trial.

Finally, the critical ratio was calculated. The various methods for calculating this have been discussed previously with different values obtained if slightly different methods are used (Wilmut *et al*., 2015). In the current study we chose to use the same method as outlined in previous papers (Wilmut & Barnett, 2011; Wilmut *et al*., 2015) in order to make comparison possible. A third‐order polynomial curve was fitted to each participant's profile of shoulder angle at the aperture across the SA ratios. The *shoulder width (SA) critical ratio* was then calculated by determining the shoulder to aperture ratio at which the shoulder angle at the door fell at one standard deviation above baseline yaw. The *body width (BA) critical ratio* was calculated by determining the body width and then re‐calculating the critical ratio in the way described. Finally, we also calculated critical ratio on the basis of body width and lateral trunk movement. The *body width + trunk movement (BTA) critical ratio* was calculated in much the same way but by taking the size of the body as being body width plus mean lateral trunk movement. All statistics were carried out in the way described in Experiment 1.

### Results

#### Critical ratio

Data for the critical ratio can be found in Table 4. A one‐way ANCOVA (group) found a significant main effect of group for the SA critical ratio [*F*(1, 55) = 16.02, *p *<* *.001, η^2^ = .23] whereby the participants with DCD showed a higher critical ratio compared to the TD participants. Age was not a significant covariate. A significant effect of group was also found for the BA ratio [*F*(1, 55) = 4.29, *p *=* *.043, η^2^ = .07], again this was due to participants with DCD showing a higher critical ratio compared to the TD participants. Age was found to be a significant covariate [*F*(1, 55) = 5.60, *p *=* *.021, η^2^ = .09]. No significant group effect was seen for the BTA critical ratio; however, age was a significant covariate [*F*(1, 55) = 6.22, *p *=* *.016, η^2^ = .10].

**Table 4:** Critical ratios: SA – shoulder width to aperture critical ratio, BA – body width to aperture critical ratio, BTA – body width and lateral trunk movement to aperture critical ratio. Standard deviation is given in parentheses

	TD	DCD
SA**	1.64 (0.10)	1.76 (0.14)
BA*	1.45 (0.11)	1.53 (0.19)
BTA	1.31 (0.09)	1.36 (0.14)

**p <* *.001; *p <* *.05.

Although the group difference was no longer apparent once lateral trunk movement had been accounted for, the participants with DCD still showed a large distribution of critical ratios. In fact, 14 children with DCD showed BTA critical ratios above the 95% confidence intervals of the TD group. It would seem, therefore, that additional factors are involved in the decision to rotate the shoulders for at least some of our participants. In previous papers it has been demonstrated that at least one of these factors is movement variability (Wilmut & Barnett, 2011; Wilmut *et al*., 2015). In order to explore this we ran correlations between shoulder angle at the aperture and our two measures of movement variability. No significant correlations were found between shoulder angle at the aperture and lateral trunk movement or shoulder angle at the aperture variability. Secondly, we ran correlations between shoulder angle at the door variability and SA and BA critical ratio. For the TD group, we found significant positive correlations between shoulder angle at the door variability and BA ratio (*r *=* *.558, *p *=* *.002) and the BTA ratio (*r *=* *.480, *p *=* *.008). For the participants with DCD significant correlations were seen for the SA ratio (*r *=* *.517, *p *=* *.004), the BA ratio (*r *=* *.633, *p *<* *.001) and the BTA ratio (*r *=* *.610, *p *<* *.001). Participants with greater movement variability showed a greater critical ratio and, therefore, rotated the shoulders to a greater degree for larger aperture ratios compared to those participants with a lesser movement variability.

#### Approach phase

**Figure 2:**
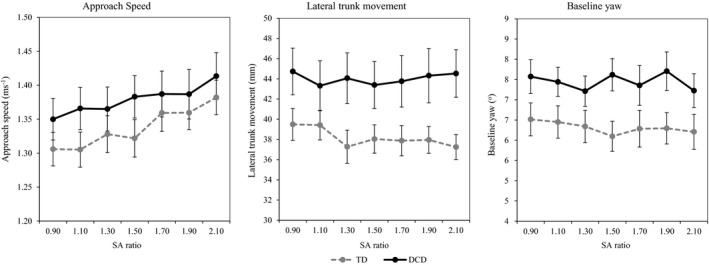
Approach values for children with and without DCD. Children with DCD are illustrated by grey dashed lines, TD children by solid black lines. Standard error is represented as error bars.

Two‐way ANCOVAs (SA ratio × group) were used to compare the approach phase variables across SA ratio and group; these data can be found in Figure 2.

For approach speed, only a significant effect of ratio was found [*F*(4.087, 224.80) = 2.93, *p *=* *.021, η^2^ = .05]. Post‐hoc tests found that this was due to lower approach speeds for the smaller compared to the larger SA ratios: 0.9 = 1.1 = 1.3 < 1.5 < 1.7 = 1.9 = 2.1. No other significant effects of group or ratio were found (*p *>* *.05). For baseline yaw and lateral trunk movement, a significant effect of group was found [*F*(1, 55) = 4.70, *p *=* *.034, η^2^ = .08, F(1, 55) = 4.97, *p *=* *.030, η^2^ = .08, respectively], with individuals with DCD showing a greater degree of yaw and a higher lateral trunk movement compared to their peers. No other significant effects of group, ratio or age were found (*p *>* *.05).

#### Crossing phase

##### Adaptations of speed

**Figure 3:**
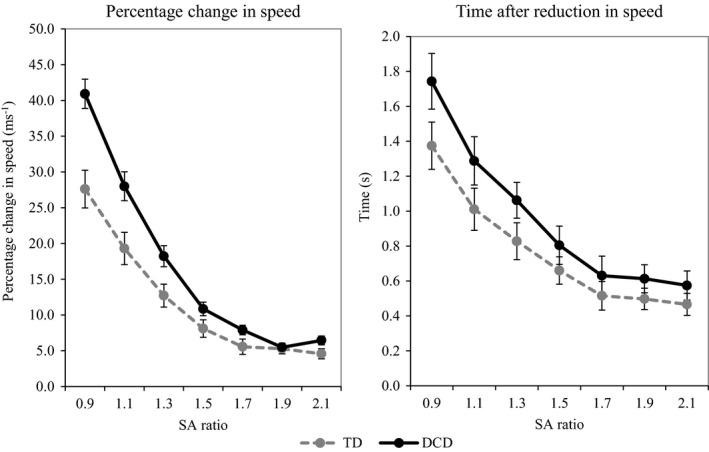
Adaptations of speed: percentage change in speed on the left and time after reduction of speed on the right. Participants with DCD are represented by a black solid line while TD participants are represented by a grey dashed line. Error bars represent standard error.

As we found approach speed to differ across the groups we calculated the percentage change in speed [(reduction in speed / approach speed)*100] which allowed us to account for this difference. Percentage change in speed was calculated for every trial – on trials where no reduction in speed was seen this was set at 0. Data can be found in Figure 3. For percentage change in speed a two‐way ANCOVA (group × SA ratio) found a significant main effect of ratio [*F*(2.349, 129.20) = 5.52, *p *=* *.001, η^2^ = .09]; post‐hoc tests showed that this was due to a higher percentage change in speed for the smaller SA ratios compared to the larger: 0.9 > 1.1 > 1.3 > 1.5 > 1.7 = 1.9 = 2.1. A significant main effect of group [*F*(1, 55) = 15.23, *p *<* *.001, η^2^ = .22] was also found, with individuals with DCD showing a higher reduction in speed compared to TD children. In addition, a significant interaction between ratio and group was found [*F*(6, 330) = 8.29, *p *<* *.001, η^2^ = .13], simple main effects showed that this was due to the participants with DCD showing a higher percentage change in speed, for the 0.9, 1.1 and 1.3 SA ratio but not for the other SA ratios (*p *<* *.05). Finally, age was found to be a significant covariate [*F*(1, 55) = 13.14, *p *=* *.001, η^2^ = .19] and to interact with group [*F*(6, 330) = 5.43, *p *<* *.001, η^2^ = .09]. This demonstrates that the influence of age on the reduction in speed at the door described above is not the same for the two groups.

Time left after the reduction in speed was only calculated for trials where we saw a reduction, and so the analyses for this variable do not include all participants (TD *N *=* *26, DCD *N *=* *27). Data can be found in Figure 3. For time left after the reduction in speed, significant main effects of ratio [*F*(3.823, 191.13) = 8.28, *p *<* *.001, η^2^ = .14], group [*F*(1, 50) = 4.27, *p *=* *.044, η^2^ = .08] and age [*F*(1, 50) = 13.107, *p *=* *.001, η^2^ = .21] were found, with the effect of ratio being due to an earlier reduction in speed for the smaller SA ratios compared to the larger: 0.9 > 1.1 > 1.3 > 1.5 = 1.7 = 1.9 = 2.1 (*p *<* *.05). The significant effect of group was due to an earlier reduction in speed by the participants with DCD compared to the TD participants. Finally, the significant effect of age demonstrates that this was a significant covariate, and age did influence the timing of the reduction in speed with an earlier reduction in speed for young children. No significant interaction between ratio and group was found (*p *>* *.05).

##### Adaptations of shoulder rotation

**Figure 4:**
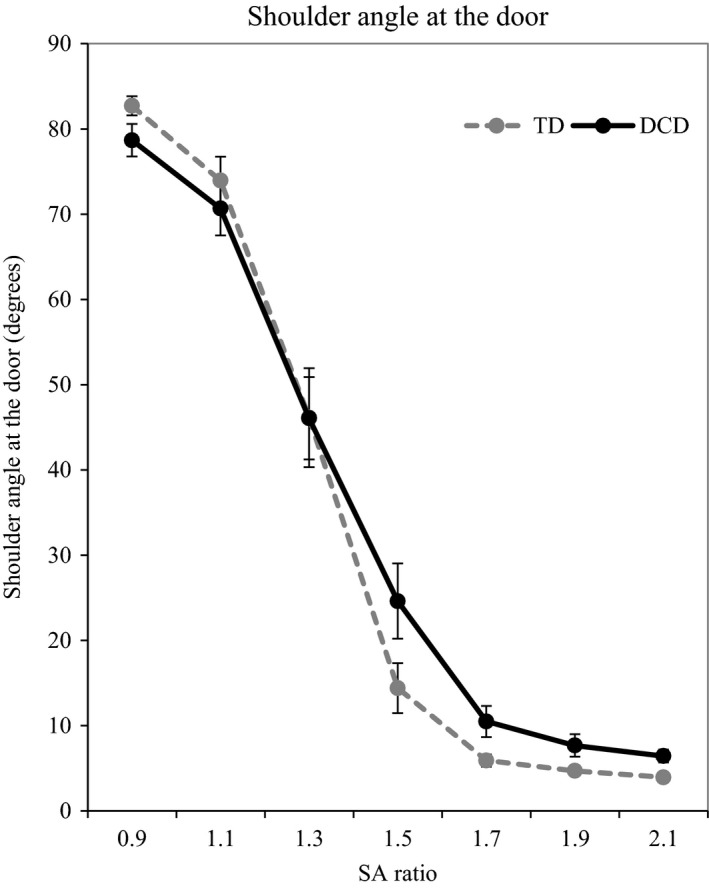
Illustration of shoulder angle at the door. Participants with DCD are represented by a black solid line, TD participants by a grey dashed line. Error bars represent standard error.

**Figure 5:**
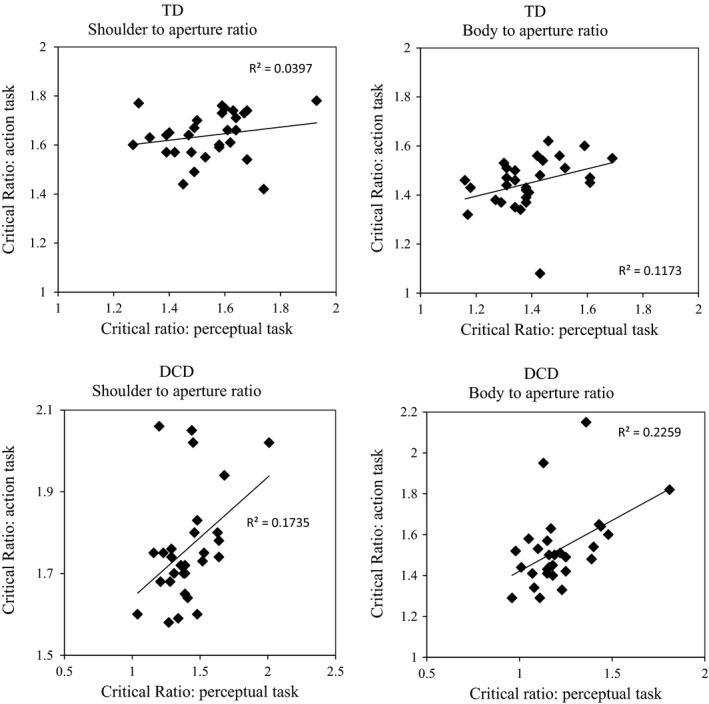
Scatter plots of critical ratios from Experiment 1 and Experiment 2. Top plots show relationships for the TD group, bottom plots show relationships for the DCD group.

For the shoulder angle at the aperture, a two‐way ANCOVA (group × SA ratio) found a significant main effect of ratio [*F*(2.299, 126.454) = 77.85, *p *<* *.001, η^2^ = .59] and a significant interaction between ratio and group [*F*(6, 330) = 3.66, *p *=* *.002, η^2^ = .06]. Data can be found in Figure 4. The main effect of SA ratio was due to significant differences in shoulder rotation at every SA ratio apart from 1.9 and 2.1: 0.9 > 1.1 > 1.3 > 1.5 > 1.7 > 1.9 = 2.1 (*p *<* *.05). For the SA ratio by group interaction, simple main effects demonstrated a significant group difference at 0.9 [*F*(1, 55) = 6.12, *p *=* *.016, η^2^ = .10], 1.5 [*F*(1, 55) = 6.68, *p *=* *.012, η^2^ = .11], 1.7 [*F*(1, 55) = 8.51, *p *=* *.005, η^2^ = .13], 1.9 [*F*(1, 55) = 9.78, *p *=* *.003, η^2^ = .15] and 2.1 [*F*(1, 55) = 15.21, *p *<* *.001, η^2^ = .22]. In all cases apart from 0.9 SA ratio, the participants with DCD showed a greater shoulder rotation compared to their peers. No significant effect of age or group was found (*p *>* *.05). Data can be found in Figure 5.

#### Relationships between Experiment 1 and Experiment 2

Correlations between the critical ratio of the perceptual action judgement seen in Experiment 1 and the critical ratio seen in this experiment were carried out on the DCD and TD group separately. Significant positive correlations were seen for the DCD group for the SA ratios (*r *=* *.419, *p *=* *.042) and the BW ratios (*r *=* *.479, *p *=* *.009). These correlations show that for the participants with DCD a high perceived SA ratio was linked to a high actual SA ratio which in turn was linked to a high shoulder rotation at the door. No significant correlations were found for the TD group. Scatter plots of these relationships can be found in Figure 4.

### Discussion

Children with DCD demonstrated a significantly larger shoulder to aperture ratio compared to their peers. This suggests that these individuals are turning for larger relatively sized apertures compared to their typically developing peers. Given that previous research studies have shown a greater incidence of raised BMI in a DCD population (Cairney *et al*., 2005), we also calculated body to aperture ratio. However, a group difference was still apparent when considering the body to aperture ratio. The final critical ratio we considered also took lateral trunk movement into account; there was no difference between the participants with and without DCD for this final critical ratio. Therefore, it seems that when scaling movements to specific aperture sizes, participants with DCD are taking both their body size and lateral trunk movement into account. However, even after this adjustment, many of the participants with DCD are showing critical ratios which fall outside the 95% confidence intervals of the TD participants, so a lack of a group difference does not necessarily mean that all participants are performing in the same way. Correlations between critical ratio and shoulder angle at the door variability may suggest that movement variability is also an important scaling factor and that it may be this that is setting some of the participants with DCD apart from the rest.

Critical ratio values seen in this study are very similar to those seen in a previous study which focused on adults with DCD and their peers (Wilmut *et al*., 2015). Although a direct analysis cannot be carried out across these data it would seem that these values do not change a great deal from childhood to adulthood in either a TD or a DCD population (in terms of shoulder to aperture ratio in the adults we found a critical ratio of 1.58 in the TD group and 1.75 in the DCD group). Furthermore, the group findings mirror those of the current study, with group differences in the shoulder to aperture ratio and in the body width to aperture ratio whereby individuals with DCD show a higher critical ratio. Wilmut *et al*. (2015) demonstrated clear correlations for the adults with DCD between lateral trunk variability and how much an individual rotates their shoulders at the aperture, and between variability in the shoulder angle at the aperture and that individual's critical ratio. In both cases adults with DCD with higher movement variability showed a greater degree of shoulder rotation at each SA ratio and also showed an overall higher critical ratio. No such relationships were seen for the TD adults. In the current study, we see a relationship between variability in the shoulder rotation at the door and critical ratio for both the children with DCD and the typically developing children. Movement in typically developing children tends to show higher variability than in adulthood (for example, see Guarrera‐Bowlby & Gentile, 2004) and so this difference may simply be due to a higher variability in the TD children compared to the TD adults in the previous study. This explanation fits with previous work which has demonstrated that young typically developing children use movement variability when scaling their body movements (Wilmut & Barnett, 2011). These correlations demonstrate that both groups of children use movement variability to scale their movements; those with a higher movement variability show a higher critical ratio, i.e. continue to rotate the shoulders for larger SA ratio than those participants with less movement variability.

In terms of other movement adaptations, the current paper has demonstrated a greater amount of baseline yaw and lateral trunk movement in the children with DCD. However, approach speed is the same for both groups. This contrasts with what was found in adults with DCD (Wilmut *et al*., 2015) who approached with a lower speed compared to the typically developing adults. Following the approach phase we considered both adaptations of speed and adaptations of shoulder rotation. The children with DCD slowed earlier in the movement and to a greater extent compared to the typically developing children for small SA ratios (0.9, 1.1 and 1.3). Furthermore, once at the aperture the children with DCD showed a greater shoulder angle for large SA ratios (1.5, 1.7, 1.9 and 2.1) and their pattern of this across SA ratios was different from the TD children. This description of behaviour during the crossing phase is in line with findings in adults with DCD (Wilmut *et al*., 2015). This seems to demonstrate a more cautious approach to both small and large apertures. When approaching a small aperture, i.e. one less than or equal to 1.3 times shoulder width, we see a larger and earlier reduction in speed. Then, when approaching a larger aperture (larger than or equal to 1.5 times shoulder width) these children no longer show a difference in the speed at which they approach but they do show a greater rotation of the shoulders. One possible explanation for this different strategy when approaching differently sized apertures may simply be due to the practical adaptations to movement that can be made. When passing through an aperture the most sensible strategy to use in order to avoid collision is increasing the safety margin, i.e. the distance between one's body and the sides of the aperture. At small aperture sizes this is not always possible as the body has a minimum medio‐lateral width. Therefore, at these small aperture sizes reducing walking speed allows for a smaller safety margin while not increasing the risk of collision. As aperture size increases, it is easy to maintain a large safety margin with a small shoulder rotation and without the need for a larger reduction in speed. This may reflect an adaptive strategy which allows children with DCD to safely pass through apertures without collision.

Given the large age range in our study, age was used as a covariate both to remove any influence it may have on the data and also to determine whether it did influence the data. Age effects were only found for the two adaptations of speed variables during the crossing phase. The younger children slowed even earlier and to a greater extent than the older children – once again this can be seen as an adaptive strategy allowing a greater amount of time in which a movement adaptation can be planned and executed.

## General discussion

In the introduction we set up the notion of a constraints‐based framework to help understand the movement difficulties of children with DCD. This posits that movements emerge from a self‐organized movement pattern that is constrained by the task, the environment and the child. Central to this is the importance of considering perception within an action context. Using this approach we considered one task (navigation through an aperture) and changed one aspect of this from Experiment 1 to Experiment 2. In Experiment 1, we considered the visual perceptual judgements of whether an aperture allowed passage in children with and without DCD. In Experiment 2, we measured movement adaptation when walking through the same apertures. This allowed us to consider how perception and action function separately and how they are related in children with and without DCD. The findings of Experiment 1 demonstrated that children with DCD show a significantly *lower* critical ratio than their peers when they are making passability judgements. In other words, a child with DCD states that they could pass through an aperture without turning while their TD counterpart states that they would need to turn. We found no accompanying visual perceptual deficit when judging absolute size in this group of children with DCD. In contrast, in Experiment 2 we found that children with DCD show a significantly *higher* critical ratio than their peers when they are actually passing through an aperture (a child with DCD turns to pass through an aperture that their TD counterpart would not turn for). These findings demonstrate the importance of considering perception within an action context. Based solely on the findings from Experiment 1, we might conclude that the perception of size is unaffected in DCD; however, when we look at this within a movement context (i.e. consideration of affordances) we see that children with DCD seem to be more accurate at these judgements, and when we add movement the pattern changes once again. This study demonstrates that perception within an action context does not reflect perception outside an action context in children with DCD. In terms of why perceptual judgements seem to be accurate while children with DCD are static (Experiment 1) but become less accurate in a dynamic context (Experiment 2), we need to consider the additional factors involved in Experiment 2. When generating a motor response, our perception of affordances is just one of the factors influencing the response we make; motor control and the ability to interpret perceptual information and generate a response is also involved. In Experiment 2 the critical ratio is the product of all of these factors and so the behaviour of children with DCD in Experiment 2 may relate more to their motor response rather than their affordance perception changing in this context.

In Experiment 2 we considered the relationship between the perceptual judgements given in Experiment 1 and the movement seen in Experiment 2. We considered this for the groups separately so that we could see how the perception–action link is different in these two populations. Intriguingly, we found positive relationships between the perceptual judgements in Experiment 1 and movement in Experiment 2 in the children with DCD but not the TD children. This finding demonstrates that if a child with DCD shows a high perceptual action critical ratio then when actually performing the movement they display this behaviour and they show a high shoulder angle at the door. This seems to describe a functional perception–action cycle. What the individual perceives in a static condition is then realized in a dynamic context. However, the TD children do not show this. In other words, the perceptual action judgements in Experiment 1 from the TD children were not related to their behaviour at the aperture in Experiment 2. This finding conflicts with Chen *et al*. (2014) who found the opposite pattern, whereby perception of sitting height was related to postural sway in the TD but not the DCD group. Therefore, our own study provides a clear link between the perception of affordances and movement within a DCD population; this is something which previously has not been demonstrated. A plausible explanation for the lack of an effect in the typically developing group may simply be a lack of variation across participants. The range of critical ratio scores was much smaller for the typically developing children and this may have precluded any significant relationships. However, central to Gibson's theory of direct perception is the importance of movement on perception, with a clear superiority of visual information that is gathered while moving as opposed to while stationary (Gibson, 1979). Therefore, it is possible that the perceptual judgements collected in Experiment 1 would typically bear no relationship to those collected in Experiment 2 given that one was in a dynamic context, while the other was in a static context. Interestingly, Warren and Whang (1987) looked at passability judgements for both a static viewing condition and a moving viewing condition (they didn't actually pass through the aperture but simply walked towards it) and they found no difference in critical ratio. This suggests that although dynamic visual information seems to result in more effective movement adaptation (Patla, 1998), this doesn't necessarily result in a more accurate perceptual judgement in typical adults (Warren & Whang, 1987). Future studies are needed to clarify this issue and to more closely consider the relationship between perception and action in children with DCD.

In conclusion, we have demonstrated that passability judgements in children with DCD under‐estimate the space they need to pass through an aperture, but that this is not due to them making equivalent movements at a doorway. Rather, it seems that perception in a static context is different from that in a dynamic context for children with DCD. However, despite this difference we have demonstrated a clear relationship between perception and action in children with DCD. We use the constraints‐based framework to advocate the need for more research which considers perception both with and without movement in order to fully understand the difficulties experienced by children with DCD.
